# (2-Bromo­phen­yl)(4-hy­droxy-1,1-dioxo-2*H*-1,2-benzothia­zin-3-yl)methanone

**DOI:** 10.1107/S1600536812013281

**Published:** 2012-03-31

**Authors:** Nazia Sattar, Hamid Latif Siddiqui, Waseeq Ahmad Siddiqui, Muhammad Akram, Masood Parvez

**Affiliations:** a Institute of Chemistry, University of the Punjab, Lahore 54590, Pakistan; b Department of Chemistry, University of Sargodha, Sargodha 40100, Pakistan; c Department of Chemistry, Universiti Teknologi Malaysia, 81310 UTM Skudai, Johor, Darul Ta’zim, Malaysia; d Department of Chemistry, The University of Calgary, 2500 University Drive NW, Calgary, Alberta, Canada T2N 1N4

## Abstract

In the title mol­ecule, C_15_H_10_BrNO_4_S, the heterocyclic thia­zine ring adopts a half-chair conformation, with the S and N atoms displaced by 0.554 (7) and 0.198 (8) Å, respectively, on opposite sides of the mean plane formed by the remaining ring atoms. The mol­ecular structure is consolidated by intra­molecular O—H⋯O inter­actions and the crystal packing features N—H⋯O and C—H⋯O hydrogen bonds.

## Related literature

For the first synthesis of benzothia­zine, see: Braun (1923 ▶). For background information on the synthesis of related compounds, see: Siddiqui *et al.* (2007 ▶). For the biological activity of 1,2-benzothia­zine derivatives, see: Lombardino & Wiseman (1972 ▶); Gupta *et al.* (1993 ▶, 2002 ▶); Zia-ur-Rehman *et al.* (2006 ▶); Ahmad *et al.* (2010 ▶). For related structures, see: Siddiqui *et al.* (2008 ▶).
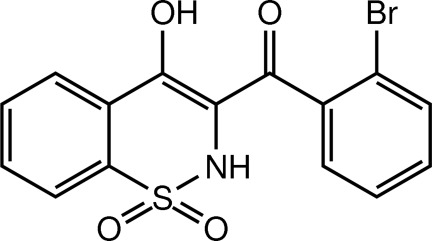


## Experimental

### 

#### Crystal data

C_15_H_10_BrNO_4_S
*M* *_r_* = 380.21
Monoclinic, 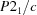

*a* = 12.0433 (4) Å
*b* = 8.5491 (3) Å
*c* = 14.7841 (5) Å
β = 106.3950 (19)°
*V* = 1460.27 (9) Å^3^
*Z* = 4
Mo *K*α radiation
μ = 2.98 mm^−1^
*T* = 173 K
0.14 × 0.12 × 0.08 mm

#### Data collection

Nonius KappaCCD diffractometer
Absorption correction: multi-scan (*SORTAV*; Blessing, 1997 ▶) *T*_min_ = 0.681, *T*_max_ = 0.797
6205 measured reflections
3339 independent reflections
2528 reflections with *I* > 2σ(*I*)
*R*_int_ = 0.043

#### Refinement

*R*[*F* ^2^ > 2σ(*F* ^2^)] = 0.048
*wR*(*F* ^2^) = 0.106
*S* = 1.10
3339 reflections
203 parameters
H atoms treated by a mixture of independent and constrained refinement
Δρ_max_ = 0.36 e Å^−3^
Δρ_min_ = −0.61 e Å^−3^

### 

Data collection: *COLLECT* (Hooft, 1998 ▶); cell refinement: *DENZO* (Otwinowski & Minor, 1997 ▶); data reduction: *SCALEPACK* (Otwinowski & Minor, 1997 ▶); program(s) used to solve structure: *SHELXS97* (Sheldrick, 2008 ▶); program(s) used to refine structure: *SHELXL97* (Sheldrick, 2008 ▶); molecular graphics: *ORTEP-3 for Windows* (Farrugia, 1997 ▶); software used to prepare material for publication: *SHELXL97*.

## Supplementary Material

Crystal structure: contains datablock(s) global, I. DOI: 10.1107/S1600536812013281/aa2054sup1.cif

Structure factors: contains datablock(s) I. DOI: 10.1107/S1600536812013281/aa2054Isup2.hkl

Supplementary material file. DOI: 10.1107/S1600536812013281/aa2054Isup3.cml

Additional supplementary materials:  crystallographic information; 3D view; checkCIF report

## Figures and Tables

**Table 1:** Hydrogen-bond geometry (Å, °)

*D*—H⋯*A*	*D*—H	H⋯*A*	*D*⋯*A*	*D*—H⋯*A*
N1—H1*N*⋯O4^i^	0.81 (5)	2.08 (5)	2.861 (4)	160 (4)
C13—H13⋯O2^ii^	0.95	2.59	3.305 (5)	132
O3—H3*O*⋯O4	0.84	1.80	2.530 (4)	145

Symmetry codes: (i) 
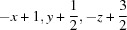
; (ii) 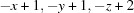.

**Fig. 1.**
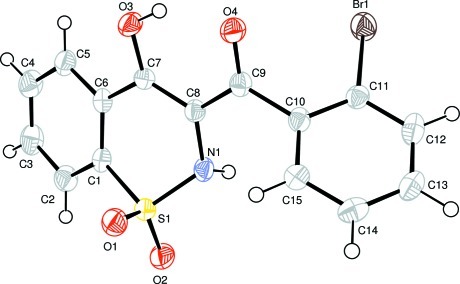
The molecular structure of the title compound with the atom numbering scheme. Displacement ellipsoids are drawn at the 50% probability level. H atoms are presented as small spheres of arbitrary radius.

**Fig. 2.**
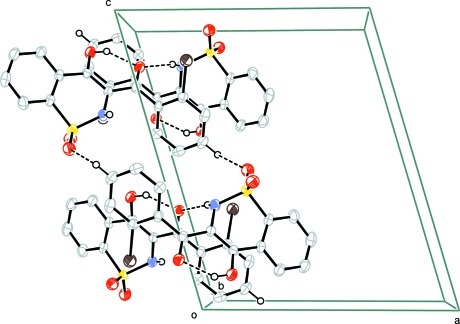
A part of the unit cell showing intermolecular and intramoilecular hydrogen bonds (dotted lines) in the crystal structure of the title compound. H atoms non-participating in hydrogen-bonding were omitted for clarity.

## supplementary crystallographic information

### Comment

Since the time first benzothiazine was synthesized (Braun, 1923), thousands of its derivatives have been prepared to determine their pharmacological and other commercial uses. Among nine isomers, the 1,2-benzothiazine 1,1-dioxide nuclei possess dynamic structural features and exhibit a wide range of biological activities, *e.g.*, anti-inflammatory (Lombardino & Wiseman, 1972), analgesic (Gupta *et al.*, 2002), anticancer (Gupta *et al.*, 1993) and antibacterial (Zia-ur-Rehman *et al.*, 2006). In continuation of our research on the synthesis of biologically active benzothiazine derivatives (Siddiqui *et al.*, 2007; Ahmad *et al.*, 2010) herein, we report the synthesis and crystal structure of the title compound.

The bond distances and angles in the title compound (Fig. 1) agree very well with the corresponding bond distances and angles reported in closely related compounds (Siddiqui *et al.*, 2008). The heterocyclic thiazine ring adopts a half chair conformation with atoms N1 and S1 displaced by 0.198 (8) and 0.554 (7) Å, respectively, on the opposite sides from the mean plane formed by the remaining ring atoms. The molecular structure is stabilized by intramolecular hydrogen bonds O3–H3O···O4 and the crystal packing is consolidated by N1—H1N···O4 and C13—H13···O2 intermolecular hydrogen bonds (Fig. 2 and Table 1).

### Experimental

A mixture of 2-[2-(*o*-bromophenyl)-2oxoethyl]-1,2-benzisothiazol-3(2*H*)-one 1,1-dioxide (1.8 g, 4.7 mmol) and sodium methoxide (1.9 g, 34.8 mmol) in freshly dried methanol (20 ml) was subjected to reflux for 30 minutes. The reaction was quenched with ice-cold water and acidified to pH = 3 with dilute HCl. The precipitate was filtered, washed with water and ethanol (25 ml, each) to get yellow powder of the title compound (1.3 g, 72%). The crystals suitable for X-ray crystallographic analysis were grown from a mixture of solvents chloroform and methanol (1:2) by slow evaporation at room temperature (m.p. 432–434 K).

### Refinement

The H atoms bonded to C and O atoms were positioned geometrically and refined using a riding model, with O—H and C—H = 0.84 and 0.95 Å, respectively. The amino H-atom was allowed to refine freely. The *U*_iso_(H) were set at 1.2*U*_eq_(parent atom).

### Crystal data

**Table:**

C_15_H_10_BrNO_4_S	*F*(000) = 760
*M**_r_* = 380.21	*D*_x_ = 1.729 Mg m^−^^3^
Monoclinic, *P*2_1_/*c*	Mo *K*α radiation, λ = 0.71073 Å
Hall symbol: -P 2ybc	Cell parameters from 3429 reflections
*a* = 12.0433 (4) Å	θ = 1.0–27.5°
*b* = 8.5491 (3) Å	µ = 2.98 mm^−^^1^
*c* = 14.7841 (5) Å	*T* = 173 K
β = 106.3950 (19)°	Prism, pale yellow
*V* = 1460.27 (9) Å^3^	0.14 × 0.12 × 0.08 mm
*Z* = 4	

### Data collection

**Table:**

Nonius KappaCCD diffractometer	3339 independent reflections
Radiation source: fine-focus sealed tube	2528 reflections with *I* > 2σ(*I*)
Graphite monochromator	*R*_int_ = 0.043
ω and φ scans	θ_max_ = 27.6°, θ_min_ = 2.8°
Absorption correction: multi-scan (*SORTAV*; Blessing, 1997)	*h* = −15→15
*T*_min_ = 0.681, *T*_max_ = 0.797	*k* = −11→10
6205 measured reflections	*l* = −19→19

### Refinement

**Table:**

Refinement on *F*^2^	Primary atom site location: structure-invariant direct methods
Least-squares matrix: full	Secondary atom site location: difference Fourier map
*R*[*F*^2^ > 2σ(*F*^2^)] = 0.048	Hydrogen site location: inferred from neighbouring sites
*wR*(*F*^2^) = 0.106	H atoms treated by a mixture of independent and constrained refinement
*S* = 1.10	*w* = 1/[σ^2^(*F*_o_^2^) + (0.0181*P*)^2^ + 4.4694*P*] where *P* = (*F*_o_^2^ + 2*F*_c_^2^)/3
3339 reflections	(Δ/σ)_max_ < 0.001
203 parameters	Δρ_max_ = 0.36 e Å^−^^3^
0 restraints	Δρ_min_ = −0.61 e Å^−^^3^

### Special details

**Table:**

Geometry. All e.s.d.'s (except the e.s.d. in the dihedral angle between two l.s. planes) are estimated using the full covariance matrix. The cell e.s.d.'s are taken into account individually in the estimation of e.s.d.'s in distances, angles and torsion angles; correlations between e.s.d.'s in cell parameters are only used when they are defined by crystal symmetry. An approximate (isotropic) treatment of cell e.s.d.'s is used for estimating e.s.d.'s involving l.s. planes.
Refinement. Refinement of *F*^2^ against ALL reflections. The weighted *R*-factor *wR* and goodness of fit *S* are based on *F*^2^, conventional *R*-factors *R* are based on *F*, with *F* set to zero for negative *F*^2^. The threshold expression of *F*^2^ > σ(*F*^2^) is used only for calculating *R*-factors(gt) *etc*. and is not relevant to the choice of reflections for refinement. *R*-factors based on *F*^2^ are statistically about twice as large as those based on *F*, and *R*- factors based on ALL data will be even larger.

### Fractional atomic coordinates and isotropic or equivalent isotropic displacement parameters (Å^2^)

**Table:**

	*x*	*y*	*z*	*U*_iso_*/*U*_eq_	
Br1	0.27922 (4)	0.40093 (6)	0.66394 (3)	0.03958 (14)	
S1	0.81353 (8)	0.40492 (13)	0.89491 (6)	0.0277 (2)	
O1	0.8507 (2)	0.2475 (4)	0.9213 (2)	0.0365 (7)	
O2	0.8381 (2)	0.5262 (4)	0.9640 (2)	0.0398 (7)	
O3	0.6691 (2)	0.2135 (4)	0.62289 (19)	0.0340 (7)	
H3O	0.6030	0.1754	0.6166	0.041*	
O4	0.4885 (2)	0.1511 (3)	0.67117 (19)	0.0317 (6)	
N1	0.6746 (3)	0.4016 (4)	0.8466 (2)	0.0269 (7)	
H1N	0.642 (4)	0.484 (5)	0.848 (3)	0.032*	
C1	0.8650 (3)	0.4564 (5)	0.7985 (3)	0.0273 (8)	
C2	0.9651 (3)	0.5437 (5)	0.8102 (3)	0.0367 (10)	
H2	1.0047	0.5867	0.8698	0.044*	
C3	1.0058 (4)	0.5666 (6)	0.7323 (3)	0.0415 (11)	
H3	1.0734	0.6279	0.7387	0.050*	
C4	0.9504 (4)	0.5020 (5)	0.6459 (3)	0.0385 (10)	
H4	0.9810	0.5178	0.5940	0.046*	
C5	0.8507 (3)	0.4147 (5)	0.6341 (3)	0.0330 (9)	
H5	0.8129	0.3709	0.5742	0.040*	
C6	0.8057 (3)	0.3911 (5)	0.7107 (3)	0.0264 (8)	
C7	0.6983 (3)	0.3029 (5)	0.6993 (3)	0.0241 (8)	
C8	0.6335 (3)	0.3124 (5)	0.7626 (3)	0.0249 (8)	
C9	0.5241 (3)	0.2322 (5)	0.7435 (3)	0.0262 (8)	
C10	0.4524 (3)	0.2456 (5)	0.8110 (3)	0.0262 (8)	
C11	0.3407 (3)	0.3076 (5)	0.7837 (3)	0.0267 (8)	
C12	0.2732 (4)	0.3111 (5)	0.8461 (3)	0.0374 (10)	
H12	0.1979	0.3557	0.8274	0.045*	
C13	0.3164 (4)	0.2495 (5)	0.9352 (3)	0.0359 (10)	
H13	0.2704	0.2511	0.9779	0.043*	
C14	0.4255 (4)	0.1857 (5)	0.9630 (3)	0.0368 (10)	
H14	0.4540	0.1419	1.0242	0.044*	
C15	0.4942 (4)	0.1852 (5)	0.9020 (3)	0.0328 (9)	
H15	0.5703	0.1433	0.9222	0.039*	

### Atomic displacement parameters (Å^2^)

**Table:**

	*U*^11^	*U*^22^	*U*^33^	*U*^12^	*U*^13^	*U*^23^
Br1	0.0341 (2)	0.0479 (3)	0.0371 (2)	0.0063 (2)	0.01057 (18)	0.0086 (2)
S1	0.0245 (5)	0.0325 (5)	0.0260 (4)	0.0008 (4)	0.0069 (4)	−0.0029 (4)
O1	0.0378 (16)	0.0376 (17)	0.0352 (15)	0.0096 (14)	0.0120 (13)	0.0072 (14)
O2	0.0340 (16)	0.0465 (19)	0.0389 (16)	−0.0021 (14)	0.0102 (13)	−0.0163 (15)
O3	0.0296 (15)	0.0432 (18)	0.0324 (14)	−0.0100 (13)	0.0142 (12)	−0.0119 (14)
O4	0.0300 (14)	0.0371 (17)	0.0300 (14)	−0.0073 (13)	0.0116 (12)	−0.0035 (13)
N1	0.0249 (16)	0.0263 (17)	0.0310 (16)	0.0043 (15)	0.0102 (13)	−0.0028 (15)
C1	0.0242 (18)	0.027 (2)	0.033 (2)	0.0018 (16)	0.0134 (16)	0.0010 (17)
C2	0.029 (2)	0.038 (2)	0.043 (2)	−0.0072 (19)	0.0109 (18)	−0.008 (2)
C3	0.032 (2)	0.042 (3)	0.057 (3)	−0.008 (2)	0.022 (2)	0.000 (2)
C4	0.035 (2)	0.044 (3)	0.042 (2)	−0.005 (2)	0.020 (2)	0.005 (2)
C5	0.032 (2)	0.036 (2)	0.035 (2)	0.0042 (19)	0.0151 (17)	0.0026 (19)
C6	0.0224 (17)	0.025 (2)	0.033 (2)	0.0017 (16)	0.0107 (15)	0.0007 (17)
C7	0.0236 (18)	0.025 (2)	0.0237 (17)	0.0011 (16)	0.0063 (14)	−0.0008 (16)
C8	0.0224 (18)	0.027 (2)	0.0261 (18)	0.0001 (16)	0.0083 (15)	0.0007 (16)
C9	0.0261 (19)	0.026 (2)	0.0285 (18)	0.0013 (16)	0.0102 (15)	0.0036 (17)
C10	0.0230 (18)	0.026 (2)	0.0315 (19)	−0.0055 (16)	0.0100 (15)	−0.0056 (17)
C11	0.0279 (19)	0.025 (2)	0.0281 (19)	−0.0016 (16)	0.0098 (16)	−0.0036 (16)
C12	0.032 (2)	0.036 (2)	0.050 (3)	0.000 (2)	0.021 (2)	−0.005 (2)
C13	0.040 (2)	0.036 (2)	0.038 (2)	−0.004 (2)	0.0227 (19)	−0.005 (2)
C14	0.046 (2)	0.040 (3)	0.026 (2)	−0.002 (2)	0.0127 (18)	0.0008 (19)
C15	0.030 (2)	0.039 (2)	0.030 (2)	0.0002 (19)	0.0095 (17)	−0.0010 (19)

### Geometric parameters (Å, º)

**Table:**

Br1—C11	1.892 (4)	C4—H4	0.9500
S1—O2	1.427 (3)	C5—C6	1.401 (5)
S1—O1	1.437 (3)	C5—H5	0.9500
S1—N1	1.623 (3)	C6—C7	1.466 (5)
S1—C1	1.763 (4)	C7—C8	1.378 (5)
O3—C7	1.327 (4)	C8—C9	1.441 (5)
O3—H3O	0.8400	C9—C10	1.498 (5)
O4—C9	1.245 (5)	C10—C11	1.395 (5)
N1—C8	1.423 (5)	C10—C15	1.395 (5)
N1—H1N	0.81 (5)	C11—C12	1.392 (5)
C1—C2	1.387 (5)	C12—C13	1.378 (6)
C1—C6	1.409 (5)	C12—H12	0.9500
C2—C3	1.386 (6)	C13—C14	1.374 (6)
C2—H2	0.9500	C13—H13	0.9500
C3—C4	1.379 (6)	C14—C15	1.385 (5)
C3—H3	0.9500	C14—H14	0.9500
C4—C5	1.382 (6)	C15—H15	0.9500
			
O2—S1—O1	120.01 (19)	O3—C7—C8	123.1 (3)
O2—S1—N1	107.91 (18)	O3—C7—C6	114.0 (3)
O1—S1—N1	107.86 (19)	C8—C7—C6	122.8 (3)
O2—S1—C1	110.4 (2)	C7—C8—N1	120.0 (3)
O1—S1—C1	107.41 (18)	C7—C8—C9	120.0 (3)
N1—S1—C1	101.73 (18)	N1—C8—C9	119.9 (3)
C7—O3—H3O	109.5	O4—C9—C8	120.5 (3)
C8—N1—S1	117.0 (3)	O4—C9—C10	119.4 (3)
C8—N1—H1N	116 (3)	C8—C9—C10	120.0 (3)
S1—N1—H1N	114 (3)	C11—C10—C15	118.4 (3)
C2—C1—C6	121.6 (4)	C11—C10—C9	121.8 (3)
C2—C1—S1	121.7 (3)	C15—C10—C9	119.7 (3)
C6—C1—S1	116.4 (3)	C12—C11—C10	120.8 (4)
C3—C2—C1	118.1 (4)	C12—C11—Br1	117.6 (3)
C3—C2—H2	120.9	C10—C11—Br1	121.5 (3)
C1—C2—H2	120.9	C13—C12—C11	119.5 (4)
C4—C3—C2	121.4 (4)	C13—C12—H12	120.3
C4—C3—H3	119.3	C11—C12—H12	120.3
C2—C3—H3	119.3	C14—C13—C12	120.6 (4)
C3—C4—C5	120.7 (4)	C14—C13—H13	119.7
C3—C4—H4	119.7	C12—C13—H13	119.7
C5—C4—H4	119.7	C13—C14—C15	120.2 (4)
C4—C5—C6	119.7 (4)	C13—C14—H14	119.9
C4—C5—H5	120.2	C15—C14—H14	119.9
C6—C5—H5	120.2	C14—C15—C10	120.5 (4)
C5—C6—C1	118.5 (4)	C14—C15—H15	119.8
C5—C6—C7	120.8 (4)	C10—C15—H15	119.8
C1—C6—C7	120.7 (3)		
			
O2—S1—N1—C8	167.0 (3)	C6—C7—C8—N1	−4.8 (6)
O1—S1—N1—C8	−61.9 (3)	O3—C7—C8—C9	−4.8 (6)
C1—S1—N1—C8	50.9 (3)	C6—C7—C8—C9	175.2 (4)
O2—S1—C1—C2	34.0 (4)	S1—N1—C8—C7	−34.1 (5)
O1—S1—C1—C2	−98.5 (4)	S1—N1—C8—C9	145.9 (3)
N1—S1—C1—C2	148.3 (4)	C7—C8—C9—O4	1.6 (6)
O2—S1—C1—C6	−152.1 (3)	N1—C8—C9—O4	−178.4 (4)
O1—S1—C1—C6	75.4 (3)	C7—C8—C9—C10	−178.5 (4)
N1—S1—C1—C6	−37.7 (3)	N1—C8—C9—C10	1.4 (6)
C6—C1—C2—C3	0.3 (6)	O4—C9—C10—C11	−60.3 (5)
S1—C1—C2—C3	173.9 (3)	C8—C9—C10—C11	119.8 (4)
C1—C2—C3—C4	−1.3 (7)	O4—C9—C10—C15	115.1 (4)
C2—C3—C4—C5	1.2 (7)	C8—C9—C10—C15	−64.7 (5)
C3—C4—C5—C6	−0.2 (7)	C15—C10—C11—C12	0.9 (6)
C4—C5—C6—C1	−0.7 (6)	C9—C10—C11—C12	176.4 (4)
C4—C5—C6—C7	178.2 (4)	C15—C10—C11—Br1	177.0 (3)
C2—C1—C6—C5	0.7 (6)	C9—C10—C11—Br1	−7.5 (5)
S1—C1—C6—C5	−173.3 (3)	C10—C11—C12—C13	−1.4 (6)
C2—C1—C6—C7	−178.2 (4)	Br1—C11—C12—C13	−177.7 (3)
S1—C1—C6—C7	7.8 (5)	C11—C12—C13—C14	0.4 (7)
C5—C6—C7—O3	18.6 (5)	C12—C13—C14—C15	1.1 (7)
C1—C6—C7—O3	−162.5 (4)	C13—C14—C15—C10	−1.6 (7)
C5—C6—C7—C8	−161.3 (4)	C11—C10—C15—C14	0.6 (6)
C1—C6—C7—C8	17.5 (6)	C9—C10—C15—C14	−175.0 (4)
O3—C7—C8—N1	175.3 (4)		

### Hydrogen-bond geometry (Å, º)

**Table:**

*D*—H···*A*	*D*—H	H···*A*	*D*···*A*	*D*—H···*A*
N1—H1*N*···O4^i^	0.81 (5)	2.08 (5)	2.861 (4)	160 (4)
C13—H13···O2^ii^	0.95	2.59	3.305 (5)	132
O3—H3*O*···O4	0.84	1.80	2.530 (4)	145

Symmetry codes: (i) −*x*+1, *y*+1/2, −*z*+3/2; (ii) −*x*+1, −*y*+1, −*z*+2.
